# APPswe mutation causes functional deficits in endothelial cells generated by transient ETV2 overexpression in human iPSCs

**DOI:** 10.1186/s12987-025-00728-8

**Published:** 2025-11-21

**Authors:** Ying-Chieh Wu, Šárka Lehtonen, Riitta Kauppinen, Hiramani Dhungana, Jari Koistinaho, Taisia Rõlova

**Affiliations:** 1 Neuroscience Center, Helsinki Institute of Life Science, University of Helsinki, Helsinki, 00014 Finland; 2 A.I.Virtanen Institute for Molecular Sciences, University of Eastern Finland, Kuopio, 70211 Finland; 3 Helsinki Institute of Life Science, University of Helsinki, Helsinki, 00014 Finland; 4 Drug Research Program, Division of Pharmacology and Pharmacotherapy, University of Helsinki, Helsinki, FI-00014 Finland

**Keywords:** Brain endothelial cells (ECs), Blood-brain barrier (BBB), Angiogenesis, Alzheimer’s disease (AD)

## Abstract

**Background:**

Brain endothelial cells (ECs) lining blood vessels are essential for the normal function of the brain. They form the first layer of the blood-brain barrier (BBB) and regulate nutrient exchange, immune responses, and angiogenesis. Numerous studies have reported the disruption of the BBB in neurodegenerative diseases, including Alzheimer’s disease (AD). However, the impact of cell-intrinsic amyloid pathology on EC function remains to be clarified.

**Methods:**

To optimize the method for producing functional ECs from human induced pluripotent stem cells (hiPSCs), we compared two different protocols. The first, a widely used method, relies on spontaneous differentiation after mesoderm specification. The second method involves transient overexpression of ETV2 to guide EC differentiation. To study the impact of beta-amyloid overproduction on EC function, we generated ECs from hiPSC lines carrying the APP Swedish mutation (APPswe), which causes AD. We assessed the functionality of both control and APPswe ECs using in vitro permeability assays, 2D and 3D vessel formation assays, and adhesion assays.

**Results:**

ECs generated using transient ETV2 overexpression exhibited higher levels of canonical EC markers, tight junction proteins, transporters, leukocyte adhesion molecules, and angiogenesis-associated receptors than ECs derived by spontaneous differentiation. Additionally, ETV2-ECs responded robustly to inflammatory and angiogenic stimuli, displaying functional and transcriptional changes, whereas spontaneously differentiated ECs did not. Consequently, we chose the ETV2 overexpression protocol to study the impact of APPswe mutation on endothelial function. We found that ETV2-ECs carrying the APPswe mutation displayed a reduced angiogenic potential following exposure to the sprouting mix and elevated expression of leukocyte adhesion molecules following inflammatory stimulation, leading to increased adhesion of monocyte-like cells.

**Conclusions:**

Overall, our study suggests that APPswe mutation in ECs impairs their response to inflammatory and angiogenic stimuli, potentially contributing to AD progression. Additionally, we confirmed that ETV2 overexpression during a critical window effectively guides hiPSCs toward the EC lineage, resulting in a stable and pure population of ECs suitable for disease modeling and drug screening.

**Supplementary Information:**

The online version contains supplementary material available at 10.1186/s12987-025-00728-8.

## Background

The brain vasculature plays a critical role in maintaining brain homeostasis. Brain endothelial cells (ECs), together with pericytes and astrocyte endfeet, form the semi-permeable blood-brain barrier (BBB), which prevents pathogens and toxins from entering the brain. The tightness of the barrier is primarily determined by tight junctions formed between ECs, while the uptake of essential nutrients from the bloodstream into the brain is mediated by specific transporters [19, 29]. In addition to their barrier functions, brain ECs also serve as major mediators of other critical processes such as immune cell adhesion and trafficking, and angiogenesis. These processes are facilitated by the leukocyte adhesion molecules (LAMs), surface proteins that regulate leukocyte attachment and transmigration across the endothelium, and by receptors responsive to angiogenic cues [15, 17, 26]. These features underscore the multifunctional role of brain ECs in vascular health and neurological function.

Numerous studies suggest that EC dysfunction contributes to the progression of neurodegenerative diseases (NDDs), including Alzheimer’s disease (AD), the most common cause of dementia. Reduced levels of tight junction proteins (TJPs), compromising barrier integrity, are a common pathological feature in brain diseases [28, 34, 46, 55]. TJP degradation permits peripheral immune cells and other harmful substances to enter the brain parenchyma, leading to neuroinflammation [18, 46]. However, other pathological changes in ECs associated with NDDs are rarely examined, leaving a significant gap in our understanding of the role of the vasculature in disease progression.

Human induced pluripotent stem cells (hiPSCs) are widely utilized in disease modeling, including BBB research. However, the existing protocols for differentiating ECs from hiPSCs have several limitations. The most-used protocol has been the one originally developed by Lippmann and coworkers [23]. This method directs cells to the mesoderm followed by spontaneous differentiation in the unconditioned medium for five days. The expression of TJPs and barrier properties are subsequently induced by adding vascular endothelial growth factor (VEGF), Wnt3, or retinoic acid [6, 24, 40]. The generated cells display relatively high transendothelial electrical resistance, active efflux transporter activity, and expression of TJPs. However, they often lack the expression of key endothelial markers such as CD31 and VE-cadherin and demonstrate limited immune responsiveness [25, 32]. Although recent protocols have addressed some of these limitations, they often depend on co-differentiation with other cell types and cell sorting, leading to variability and batch-to-batch inconsistency [32, 39]. Therefore, simpler and more effective methods are needed to generate hiPSC-derived ECs that consistently retain endothelial characteristics and in vivo-like functions.

E26 transformation-specific (ETS) variant 2 (ETV2) is a member of the ETS transcription factor family, characterized by a DNA-binding domain that recognizes specific DNA sequences to regulate gene expression. ETV2 is a pivotal regulator of endothelial lineage commitment, essential for the differentiation of ECs during embryonic development [13, 20, 45]. It guides hiPSCs toward endothelial fate by upregulating a set of genes critical for EC identity and function, including *TEK*, *KDR*, *PECAM1*, and *NOS3*, which are involved in angiogenesis and vascular integrity [13, 20, 33]. Several studies have employed ETV2 to drive endothelial differentiation from hiPSCs using diverse methods [31, 53, 58]. However, the potential of ECs generated using ETV2 overexpression (ETV2-ECs) to model NDD pathology has not been thoroughly investigated.

To validate that ETV2 expression can robustly guide hiPSC differentiation toward an EC fate and produce ECs with in vivo-like properties, we transduced three hiPSC lines with ETV2 under a doxycycline-inducible promoter, allowing overexpression during critical developmental windows. For comparison, we used another widely referenced protocol by Lippmann and coworkers to generate ECs [23, 24], referred to as spontaneously derived ECs (S-ECs). While we were unable to generate functional ECs using the spontaneous protocol, ETV2-ECs expressed high levels of EC markers and responded to inflammatory and angiogenic stimuli, confirming their potential as a model for studying immune responses and angiogenesis. To investigate the impact of amyloid pathology, we introduced ETV2 into three lines carrying the KM670/671NL mutation, known as the APP Swedish mutation (APPswe), which causes familial AD [43]. Our findings showed that APPswe ECs expressed higher levels of LAMs after inflammatory stimulation and exhibited impaired angiogenic responses compared to control ECs. Overall, these results suggest that overexpression of ETV2 in hiPSCs is an effective approach to generate ECs suitable for modeling NDDs.

## Materials and methods

### Generation of ETV2-overexpressing HiPSCs

The hiPSC lines used in this study are summarized in Table 1. All original hiPSC lines expressed characteristic pluripotency markers, maintained normal karyotypes, and demonstrated differentiation potential into embryoid bodies comprising all three germ layers. The lines were maintained on Matrigel (growth factor reduced; Corning; 1:200)-coated 3.5 cm cell culture dishes (Sarstedt) in Essential 8 (E8) Medium (Gibco, Thermo Fisher Scientific) and passaged using 0.5 mM EDTA (Thermo Fisher Scientific) every 4–5 days. All cell culturing was done at 37 °C and 5% CO_2_.

The doxycycline-inducible ETV2 transgene was introduced into hiPSCs via the lentiviral vector pInducer20-ETV2 as described [54]. The plasmid was generated by Gateway cloning at the Genome Biology Unit, University of Helsinki, using ETV2 clone (GenBank: BC160032.1) in pENTR223.1 vector and pInducer20 (Addgene, # 44012) as a backbone. The plasmid was packaged into second-generation lentiviral particles at the Biomedicum Virus Core, University of Helsinki. Following ETV2 transduction, the modified cell lines underwent additional validation to confirm stable karyotypes and effective induction of ETV2 expression (Figure S1A–B). The relative *ETV2* copy number was further assessed using genomic DNA by qPCR using CFX96 Real-Time PCR System (Bio-Rad). Genomic DNA was extracted using the NucleoSpin Tissue XS (Macherey-Nagel), following the manufacturer’s instructions. qPCR was performed with primers targeting the *ETV2* transgene and the *WPRE* (woodchuck posttranscriptional regulatory element) sequence present in the viral vector. Non-transduced cells, which contain only endogenous *ETV2* (two copies per diploid genome) and no *WPRE*, were used as the reference for normalization. Human *GAPDH* was used as a single-copy reference gene. Ct values for *ETV2* and WPRE were first normalized to *GAPDH*, and relative transgene abundance across transduced lines was then compared and expressed as fold change relative to the Ctrl1-ETV2 line. In non-transduced lines, *ETV2* levels were very low and *WPRE* was undetectable, confirming their use as negative controls. Probe and primer sequences for *WRPE* were used as described [38]: Probe: 6-FAM 5’-TGCTGACGCAACCCCCACTGGT-3’TAMARA, forward primer: 5’-CCGTTGTCAGGCAACGTG-3’, reverse primer: 5’-AGCTGACAGGTGGTGGCAAT-3’(Merck).

The relative *ETV2* copy number ranged from 0.54- to 2.57-fold compared to Ctrl1-ETV2. *WPRE* showed a similar fold-change pattern, further supporting the *ETV2* copy number estimates (Figure S1 C-D).

**Table 1:** Human HiPSC lines used in this study

Represent symbol	Cell line	Sex	Age at Biopsy	APP Genotype	APOE Genotype	Status When Sample Taken	References
	Ctrl1	F	77	Control	ɛ3/ɛ3	Healthy	[54]
	Ctrl1-ETV2						Figure S1.
	Ctrl2	F	77	Control	ɛ3/ɛ3	Healthy	[54]
	Ctrl2-ETV2						Figure S1.
	Ctrl3	M	64	Control	ɛ3/ɛ3	Healthy	[54]
	Ctrl3-ETV2						Figure S1.
	AD1	F	58	APPswe	ɛ3/ɛ3	AD	[35]
	AD1-ETV2						Figure S1.
	AD2	F	30	APPswe	ɛ3/ɛ3	Pre-symptomatic AD	[22]
	AD2-ETV2						Figure S1.
	AD3	M	15–19	APPswe	ɛ3/ɛ4	Healthy control introduced APPswe	[12]
	AD3-ETV2						Figure S1.

### Differentiation of ETV2-ECs

The differentiation protocol for generating ETV2-ECs was adapted based on the method by Wang and colleagues [53], with certain modifications detailed previously [54]. Briefly, hiPSCs expressing ETV2 were dissociated and seeded at a density of 2.2 × 10⁴ cells/cm² in E8 medium supplemented with ROCK inhibitor Y-27,632. On day 1, the medium was switched to a DMEM/F12-based formulation (Thermo Fisher Scientific) containing 6 µM CHIR99021 (Cayman Chemical), maintained for two days. On day 3, the cells were transitioned into StemPro-34 SFM medium (Thermo Fisher Scientific) supplemented with VEGF-A (50 ng/ml), basic fibroblast growth factor (bFGF; 50 ng/ml), epidermal growth factor (EGF; 10 ng/ml) from PeproTech (Thermo Fisher Scientific), SB431542 (10 µM), and doxycycline hyclate (2 µM; BioGems), cultured for two additional days. On day 5, the medium was changed to Human Endothelial SFM supplemented with 5% KnockOut Serum Replacement (Thermo Fisher Scientific), bFGF (10 ng/ml), EGF (5 ng/ml), and VEGF (0.5 ng/ml). The cells were maintained and routinely passaged in this medium for up to one week prior to experimentation.

### Differentiation of S-ECs

The S-ECs differentiation protocol was adapted from Blanchard et al. [6, 40]. On day 0, hiPSCs were dissociated into single cells using StemPro Accutase (Thermo Fisher Scientific) and replated on Matrigel-coated plates at a density of 5.5 × 10³ cells/cm² in E8 medium supplemented with 10 µM ROCK inhibitor Y-27,632. On day 1, the medium was switched to S1 medium (DMEM/F12 with 1× GlutaMAX, 50 µM 2-mercaptoethanol, 1× MEM-NEAA, and 0.5% penicillin/streptomycin (all from Gibco, Thermo Fisher Scientific)) supplemented with 6 µM CHIR99021 for 2 days. On day 3, the medium was replaced with S2 medium (S1 medium supplemented with 2% B-27 (Gibco)), which was refreshed daily for the next 5 days. After five days in S2 medium, the cells were transitioned to endothelial medium (Human Endothelial SFM (Thermo Fisher Scientific) with 2% B-27) supplemented with 10 µM retinoic acid (RA, Sigma) and 20 ng/ml bFGF (PeproTech, Thermo Fisher Scientific). The resulting S-ECs were maintained and passaged using Accutase in endothelial medium until use.

### Differentiation of pericyte-like cells

The protocol for differentiating hiPSCs into pericyte-like cells was adapted from the methods described by Blanchard and colleagues [6], incorporating minor adjustments detailed previously [54]. Briefly, hiPSCs were dissociated into single-cell suspensions and seeded in Essential 8 medium supplemented with ROCK inhibitor Y-27,632. From days 1 to 4, cells were cultured in a medium composed of DMEM/F12 and Neurobasal (Thermo Fisher Scientific), supplemented with 25 ng/mL bone morphogenetic protein-4 (BMP-4; PeproTech, Thermo Fisher Scientific) and 8 µM CHIR99021. On days 5 and 6, the medium was switched to one containing 10 ng/ml platelet-derived growth factor-BB (PDGF-BB) and 2 ng/ml transforming growth factor-beta 3 (TGFβ-3; both from PeproTech, Thermo Fisher Scientific). The pericyte-like cells obtained through this protocol were cultured for an additional two weeks prior to experimentation.

### Immunocytochemistry

Cells were washed with PBS and fixed using 3.7% formaldehyde (Merck) for 20 min at room temperature (RT). Subsequently, the cells were permeabilized with 0.3% Triton X-100 (Merck) and then incubated in a blocking solution containing 5% normal goat serum (NGS; Merck) for 1 h at RT. Primary antibodies diluted in PBS containing 5% NGS were applied overnight at 4 °C, followed by incubation with secondary antibodies for 1 h at RT. Nuclei were counterstained using 1 µg/ml DAPI (Sigma) for 10 min at RT. After staining, coverslips were mounted using Fluoromount-G™ mounting medium (Thermo Fisher Scientific), and images were acquired using either the EVOS M5000 Imaging System (Thermo Fisher Scientific) with 4×, 10× or 20× objectives or LSM 710 confocal microscope (Zeiss) with a 40x objective. Details of primary and secondary antibodies are provided in Table 2.

ZO-1 intensity quantification before and after cytokine exposure was performed using images acquired using EVOS Imaging System with 20× objective. For each cell line per batch, two fields per slide were captured from two slides. Integrated density and nuclei count were measured using FIJI ImageJ, and mean fluorescence intensity per cell was calculated by dividing integrated density by cell number.

**Table 2:** Primary and secondary antibodies used for immunocytochemistry

Target	Dilution	Source	Catalog no.
Rabbit anti-VE-cadherin	1:500	abcam	ab232880
Mouse anti-CD31	1:500	Agilent Dako	M0823
Rabbit anti-ZO-1	1:200	Rockland, Thermo Fisher Scientific	600-401-GU7
Goat anti-Mouse IgG (H + L), Alexa Fluor™ 488	1:300	Thermo Fisher Scientific	A-11,001
Goat anti-Rabbit IgG (H + L), Alexa Fluor™ 568	1:300	Thermo Fisher Scientific	A-11,011
Goat anti-Rabbit IgG (H + L), Alexa Fluor™ 488	1:300	Thermo Fisher Scientific	A-11,008

### Quantitative real-time PCR (qPCR)

Total RNA was isolated from hiPSCs, ETV2-ECs, S-ECs, pericyte-like cells, and MLCs using the RNeasy Mini Kit (Qiagen), adhering to the manufacturer’s recommended protocol. RNA concentrations were quantified with a SimpliNano Spectrophotometer (Biochrom). Complementary DNA (cDNA) synthesis was carried out using the Maxima Reverse Transcriptase Kit supplemented with RiboLock RNase Inhibitor, dNTP Mix, and Random Hexamer Primer (all reagents sourced from Thermo Fisher Scientific). qPCR was performed to measure mRNA expression utilizing TaqMan assay probes (listed in Table 3), along with Maxima Probe/ROX qPCR Master Mix (Thermo Fisher Scientific) on a CFX96 Real-Time PCR System (Bio-Rad). Gene expression values were normalized against *GAPDH*.

**Table 3:** Primers assay mixes used for mRNA expression studies

Gene	Identifier	Source
*PECAM1*	Hs00169777_m1	TaqMan, Thermo Fisher Scientific
*CDH5*	Hs00901465_m1	TaqMan, Thermo Fisher Scientific
*VWF*	Hs01109446_m1	TaqMan, Thermo Fisher Scientific
*TJP1*	Hs01551871_m1	TaqMan, Thermo Fisher Scientific
*OCLN*	Hs05465837_g1	TaqMan, Thermo Fisher Scientific
*ABCC1*	Hs01561483_m1	TaqMan, Thermo Fisher Scientific
*ABCG2*	Hs01053790_m1	TaqMan, Thermo Fisher Scientific
*CDH1*	Hs01023895_m1	TaqMan, Thermo Fisher Scientific
*KRT8*	Hs01595539_g1	TaqMan, Thermo Fisher Scientific
*KRT14*	Hs00265033_m1	TaqMan, Thermo Fisher Scientific
*ICAM1*	Hs00164932_m1	TaqMan, Thermo Fisher Scientific
*VCAM1*	Hs01003372_m1	TaqMan, Thermo Fisher Scientific
*KDR*	Hs00911700_m1	TaqMan, Thermo Fisher Scientific
*S1PR1*	Hs05021992_s1	TaqMan, Thermo Fisher Scientific
*ITGAL*	Hs00158218_m1	TaqMan, Thermo Fisher Scientific
*ITGAM*	Hs00167304_m1	TaqMan, Thermo Fisher Scientific
*ETV2*	Hs01012852_g1	TaqMan, Thermo Fisher Scientific
*GAPDH*	Hs99999905-m1	TaqMan, Thermo Fisher Scientific

### Permeability assay

ECs were plated onto the basolateral surface of Transwell inserts (Corning; 0.4 μm pore size), pre-coated with Matrigel, at a density of 5 × 10⁵ cells/cm² in endothelial cell medium containing 10 µM ROCK inhibitor Y-27,632. After an initial 24-hour culture period, half of the culture medium was refreshed, with partial medium changes performed every two days until day 4. On day 5, cells were stimulated with 20 ng/ml TNFα and 20 ng/ml IL-1β (both from PeproTech, Thermo Fisher Scientific), or left untreated as control conditions. After an additional 24 h, a permeability assay was conducted. To perform this assay, Alexa 488-conjugated dextran (4 kDa; Sigma) was added to the medium to a final concentration of 0.5 mg/ml. A standard curve was prepared using serial 5-fold dilutions ranging from 0.5 mg/ml down to 160 ng/ml. For the permeability measurement, 900 µl of fresh medium was placed into the lower chamber, while 300 µl of dextran-medium mixture was added to the upper chamber. After incubation at 37 °C for one hour, 100 µl of medium from the lower chamber was collected and fluorescence intensity was measured using a FLUOstar Omega spectrometer (BMG Labtech).

### Generation of TdTomato-expressing MLCs

The Ctrl3 hiPSC line was plated on a Matrigel-coated 3.5 cm plate in E8 medium. The following day, the old medium was removed, and cells were transduced with LV-CMV-TdTomato-puro lentivirus (SignaGen Laboratories, replication-competent lentivirus titer negative, endotoxin negative) at a concentration of 3,556 TU/µl in 900 µl of E8 medium supplemented with 5 µM Y-27,632. One-hour post-transduction, 600 µl of fresh E8 medium was added to the cells. The next day, the medium was completely replaced with fresh E8 medium. 48 h after transduction, the culture medium was switched to E8 medium containing 0.4 µg/ml puromycin (Fisher Scientific) to eliminate non-transduced cells. After 14 days, cell sorting was performed using Sony SH800Z Cell Sorter at the Biomedicum Flow Cytometry core facility, University of Helsinki, to isolate clones exhibiting a specific fluorescence intensity.

For the monocyte differentiation, hiPSCs were first differentiated into hematopoietic progenitor cells using the StemDiff Hematopoietic Kit (StemCell Technologies) according to the manufacturer’s instructions. Hematopoietic progenitors were further differentiated for 5 days towards myeloid lineage using the medium containing DMEM/F12, 2× insulin-transferrin-selenite, 2× B27, 0.5× N2, 1× GlutaMAX, 1× MEM-NEAA (all from Thermo Fisher Scientific), 400 µM monothioglycerol (Merck), 5 µg/ml human insulin (Merck), 100 ng/ml human IL-34, 50 ng/ml human TGF-β1, and 25 ng/ml human M-CSF (all cytokines from Peprotech, Thermo Fisher Scientific) with half-media change performed every other day as described [1, 16]. MLCs were then thawed 2 days before the assay in MLC medium (RPMI medium supplemented with 20 ng/ml IL-6, 20 ng/ml IL-3, 50 ng/ml SCF (all from PeproTech, Thermo Fisher Scientific) and 10% FBS (Thermo Fisher Scientific)) for recovery.

### MLC adhesion to ECs

ECs were replated at a density of 1 × 10⁵ cells/cm² in EC culture medium on Matrigel-coated 24-well plates. The following day, ECs were treated with 20 ng/ml TNFα and 20 ng/ml IL-1β for 24 h. On the assay day, MLCs were pre-treated with 100 ng/ml Phorbol 12-myristate 13-acetate (PMA, Thermo Fisher Scientific) for 4 h. At the start of the adhesion assay, the old medium was removed from the EC plate, and 2 × 10⁴ MLCs/well were added in a 1:1 mixture of EC and MLC medium onto the EC layer. The plate was placed on an orbital shaker at 60 rpm for 40 min at 37 °C for adhesion. After incubation, the wells were washed three times with DPBS to remove unbound MLCs. Nuclei were stained with 1 µg/ml Hoechst for 10 min at RT. Images were captured using the EVOS imaging system with a 4× objective, utilizing red and blue fluorescent channels. For quantification, MLCs and nuclei were counted from three fields per well, and MLC adhesion was expressed as the ratio of MLCs to total nuclei staining for normalization.

### 2D tube formation assay

A 96-well plate was coated with 50 µl of Matrigel and incubated for at least 30 min to ensure polymerization. ECs were prepared at a density of 3 × 10⁴ cells per well and labeled with 100 nM Calcein AM (Cayman Chemical) for 15 min at 37 °C. The fluorescently labeled ECs were seeded onto the polymerized Matrigel in either standard EC culture medium (control condition) or EC medium supplemented with a sprouting mix containing 3 ng/ml vascular endothelial growth factor (VEGF), 5 ng/ml basic fibroblast growth factor (bFGF), 3 ng/ml phorbol 12-myristate 13-acetate (PMA), and 30 nM sphingosine-1-phosphate (S1P; Sigma). Images were acquired every 2 h using an Incucyte S3 system (Sartorius) (located at the Biomedicum Stem Cell Center, University of Helsinki) equipped with a whole-well imaging module, utilizing 4× magnification with both phase contrast and green fluorescence channels. For quantitative analysis, images captured at 6 h post-seeding were processed using the FIJI ImageJ Angiogenesis Analyzer module (Carpentier et al., 2020) to measure parameters including segment count, segment length, mesh number, and mesh area.

### 3D vessel sprouting assay

The 3D vessel sprouting protocol was adapted from [48] with modifications. ECs were plated in an ultra-low attachment 6-well plate (Corning) at a density of 6 × 10⁵ cells/well in EC medium and cultured for 24 h to allow aggregate formation. After 24 h, the aggregates were collected into a 15 ml tube, allowed to settle at room temperature for 15 min, and then resuspended in 300 µl of Matrigel. The aggregates were then embedded in Matrigel and seeded into a 24-well plate. The Matrigel was either left untreated (control) or supplemented with a sprouting mix containing 3 ng/ml VEGF, 5 ng/ml bFGF, 3 ng/ml PMA, and 30 nM S1P. The plate was incubated at 37 °C for 1 h to allow Matrigel polymerization. Following polymerization, EC medium (either untreated or supplemented with the sprouting mix) was added on top of the Matrigel layer. After 24 h, images were captured using the EVOS imaging system with a 10× objective to observe vessel sprouting. For quantification, the perimeter of each aggregate was measured, and the number of sprouting ends was counted. The final data were normalized to the perimeter, and results were expressed as the number of sprouting ends per 100 μm.

### Statistics

Statistical analysis was performed using GraphPad Prism 10 software (GraphPad Software Inc.). Differences between two groups were assessed using Student’s t-test. Comparisons across multiple groups were evaluated by one-way ANOVA with Dunnett’s multiple comparison test. For experiments involving two independent variables, a two-way ANOVA with Bonferroni’s multiple comparison test was utilized. Statistical significance was defined as *p* < 0.05. Data in figures are presented as mean ± SD.

## Results

### ETV2-ECs exhibit stronger endothelial identity compared to S-ECs

**Fig. 1:**
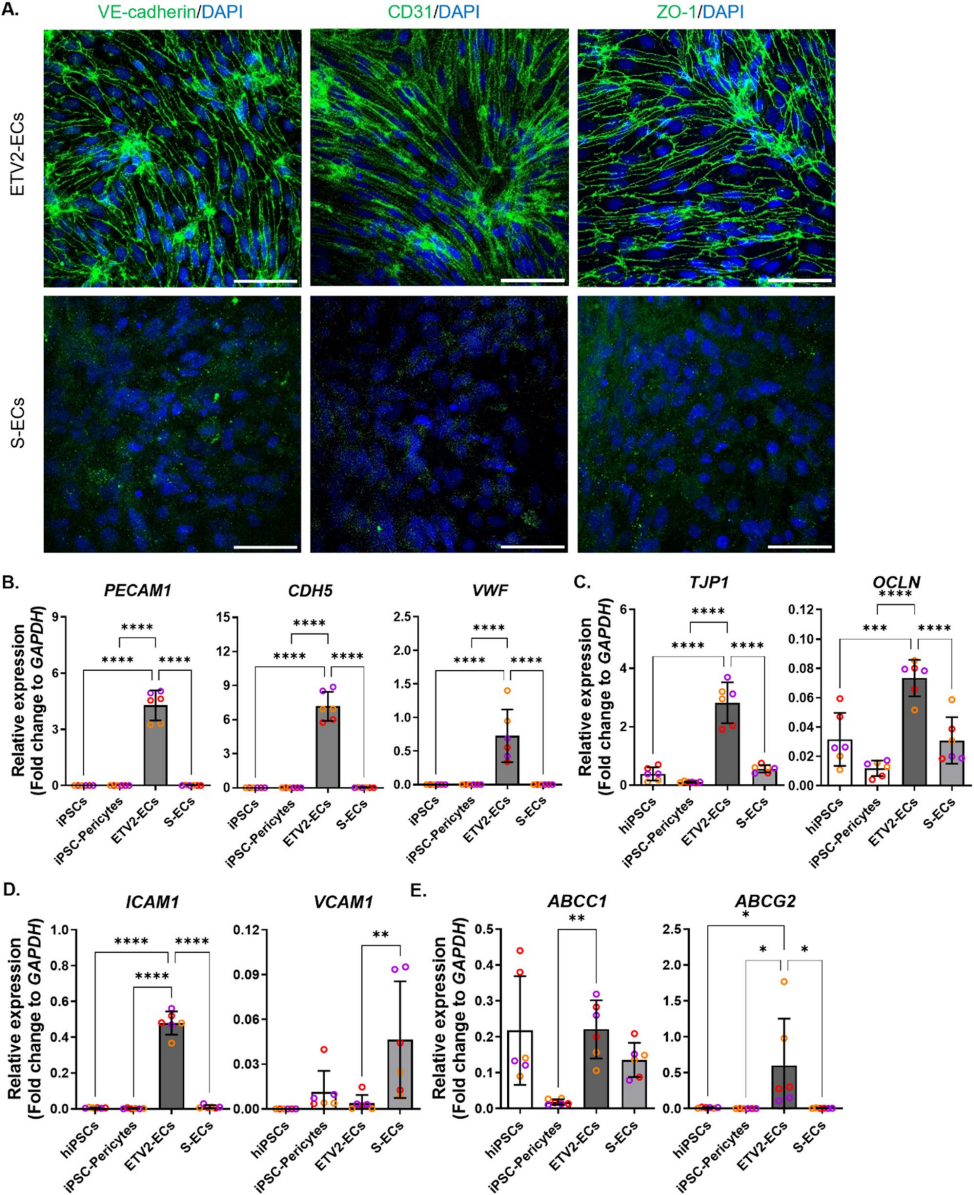
Characterization of ETV2 and S-endothelial cells (ECs). (**A**) Immunostaining for VE-cadherin, CD31, and ZO-1 in ETV2 and S-ECs derived from control lines. Nuclei are counterstained with DAPI. Scale bars, 50 μm. (**B**-**F**) Relative mRNA levels of *PECAM1*, *CDH5*, *VWF* (**B**), *TJP1*, *OCLN* (**C**), *ICAM1*, *VCAM1* (**D**), and *ABCC1*, *ABCG2* (**E**) in hiPSC-pericytes, S-ECs, ETV2-ECs, and hiPSCs, shown as fold change relative to *GAPDH*. Dot plots represent the average of technical replicates for each differentiation batch, color-coded by hiPSC line. Data are presented as mean ± SD. Statistical significance was assessed using one-way ANOVA with Dunnett’s multiple comparison test, indicated as *p < 0.05, **p < 0.01, ***p < 0.001, ****p < 0.0001

Following differentiation, the identities of cells generated using two different protocols, ETV2 and spontaneous, were thoroughly assessed. Immunocytochemistry confirmed the expression of classic EC markers VE-cadherin and CD31, along with TJP ZO-1 in ETV2-ECs (Fig. 1A). In contrast, S-ECs did not exhibit VE-cadherin and CD31 immunoreactivity and displayed very low levels of ZO-1 in the cytosol. qRT-PCR (qPCR) further confirmed that *PECAM1* and *CDH5*, encoding for CD31 and VE-cadherin, respectively, were significantly upregulated in ETV2-ECs compared to hiPSCs, hiPSC-derived pericyte-like cells, and S-ECs (Fig. 1B). Similarly, Von Willebrand Factor (VWF), critical for hemostasis and vascular integrity, showed high expression in ETV2-ECs but was absent in S-ECs (Fig. 1B). Consistent with immunocytochemical results, TJPs including *TJP1* (ZO-1) and *OCLN* (Occludin), essential for maintaining endothelial barrier integrity, were also significantly elevated in ETV2-ECs compared to S-ECs (Fig. 1C).

LAMs, important for immune cell trafficking, showed differential expression: *ICAM1* (Intercellular Adhesion Molecule 1) was strongly elevated in ETV2-ECs while *VCAM1*(Vascular cell adhesion protein 1) was moderately increased in S-ECs (Fig. 1D). Additionally, specific efflux transporters were analyzed. *ABCC1* (Multidrug Resistance Protein 1, MRP1) levels were comparable between the ECs from two protocols, whereas *ABCG2* (Breast Cancer Resistance Protein, BCRP) was notably higher in ETV2-ECs (Fig. 1E).

These findings indicate that ETV2-ECs exhibit enhanced expression of classical endothelial markers and genes linked to brain EC functions, such as TJPs, LAMs, and angiogenesis-related genes, compared to S-ECs, epithelial cells and pericytes.

### In contrast to S-ECs, ETV2-ECs showed increased permeability and reduced TJP expression after a pro-inflammatory cytokine exposure

**Fig. 2:**
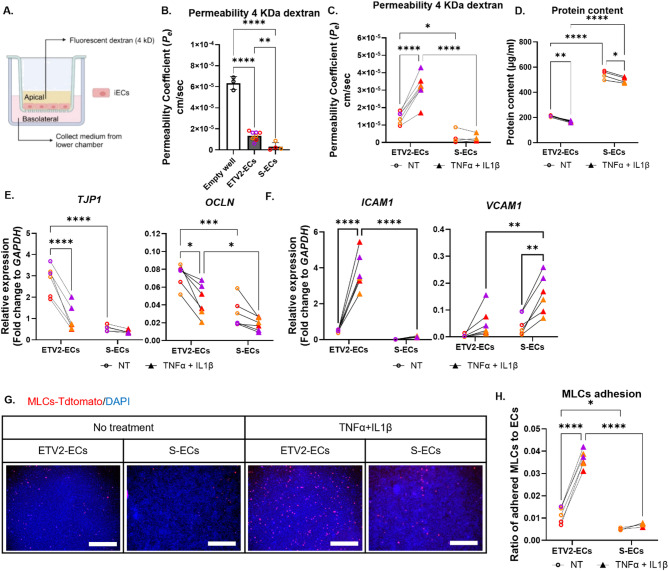
ETV2-ECs reacted to challenges with pro-inflammatory cytokines by exhibiting functional changes, while S-ECs did not show similar responses. (**A**) Schematic of the experimental setup for permeability assays. (**B**) Measurement of endothelial permeability coefficients (Pe) using 4 kDa fluorescently labeled dextran in S-ECs, ETV2-ECs, and empty wells (controls). (**C**) Permeability of the 4 kDa fluorescently labeled dextran in S and ETV2-ECs before and after exposure to TNFα and IL1β. (**D**) Total protein content measured post-assay as an indicator of cell density. (**E**-**F**) Relative mRNA levels of *TJP1*,* OCLN* (**E**), *ICAM1*,* VCAM1* (**F**) in S and ETV2-ECs before and after cytokine exposure, normalized to *GAPDH* and expressed as fold change. (**G**) Representative images of DAPI-stained cells after the adhesion assay; red signal indicates monocyte-like cells (MLCs) adhered to S-ECs or ETV2-ECs following washing. Scale bars, 750 μm. (**H**) Quantification of adhered MLCs, calculated as the ratio of red MLCs to total DAPI-stained nuclei per field. Dot plots represent the average of technical replicates for each differentiation batch, color-coded by hiPSC line. Data are presented as mean ± SD. Statistical analysis used one-way ANOVA with Dunnett’s multiple comparison test for (**B**), and two-way ANOVA with Bonferroni’s multiple comparison test (C-F, H). Significance levels are marked as *p < 0.05, **p < 0.01, ***p < 0.001, ****p < 0.0001

Brain ECs preserve barrier integrity, which is necessary for homeostasis and correlates with vascular health. We used a Transwell permeability assay to confirm that the ECs formed a barrier (Fig. 2A). Both ETV2-ECs and S-ECs demonstrated decreased permeability when compared to empty wells, with S-ECs showing even lower permeability than ETV2-ECs (Fig. 2B), despite ETV2-ECs having higher TJP expression (Fig. 1C). Following the permeability assay, we lysed cells from the inserts and discovered that the protein concentration in S-EC lysates was three times higher than in ETV2-ECs, suggesting that S-ECs proliferated and/or survived better (Fig. 2D). This difference might explain the lower permeability observed in S-EC cultures. Since inflammation-induced barrier disruption is frequently observed in NDDs, we evaluated the EC responsiveness to pro-inflammatory stimuli to determine whether they could serve as disease models. Our data revealed that, unlike ETV2-ECs, S-ECs showed no significant increase in permeability when exposed to tumor necrosis factor alpha (TNFα) and interleukin-1 beta (IL1β) (Fig. 2C). TNFα and IL1β exposure decreased protein concentration in the lysates of both cell types, indicating cell loss due to the inflammatory challenge (Fig. 2D). We further examined the changes in TJPs by qPCR and found that *TJP1* and *OCLN* expression was significantly decreased in ETV2-ECs after exposure to TNFα and IL1β, while only a minor reduction was seen in S-ECs (Fig. 2E). Also, *VWF* expression significantly decreased after exposure in ETV2-ECs, with no changes noted in S-ECs (Figure S2 A). The inflammatory exposure did not cause any significant changes in the expression of basal endothelial markers *PECAM1* or *CDH5* in either S-ECs or ETV2-ECs.

### The adhesion of monocyte-like cells (MLCs) to ETV2-ECs increased following exposure to TNFα and IL1β

We previously identified baseline differences in *ICAM1* and *VCAM1* levels between ETV2-ECs and S-ECs (Fig. 1D). These molecules are LAMs essential for immune cell adhesion to ECs and subsequent trafficking under inflammatory conditions. TNFα and IL1β exposure increased *ICAM1* expression in both ETV2 and S-ECs approximately 10-fold. However, baseline and post-exposure levels were about 40 and 25 times greater in ETV2-ECs than in S-ECs (Fig. 2F). Conversely, *VCAM1* expression was higher in S-ECs compared to ETV2-ECs at baseline and after cytokine exposure, but only by 10-fold before and 3-fold after exposure to TNFα and IL1β (Fig. 2F). To investigate the biological role of the upregulated LAMs, we performed an adhesion assay using PMA-pretreated hiPSC-derived MLCs. MLCs adhered significantly stronger to ETV2-ECs exposed to TNFα and IL1β compared to unexposed ECs. However, no significant increase in adhesion was found in S-ECs (Fig. 2G-H). The qPCR analysis of MLCs confirmed that PMA increased expression of CD11a (*ITGAL*) and CD11b (*ITGAM*), which pair with CD18 to form the integrins LFA-1 and Mac-1, respectively—both known ligands for ICAM1 (Yang et al., 2007; Dustin and Springer, 1988) (Figure S2 B). Combined with the permeability assay data, these findings indicate that ETV2-ECs respond to inflammatory challenges by exhibiting changes at both the transcriptional and functional levels after cytokine exposure, which were not observed in S-ECs.

### ETV2-ECs can respond to angiogenic stimuli by forming vessel-like structures in both 2D and 3D models

**Fig. 3:**
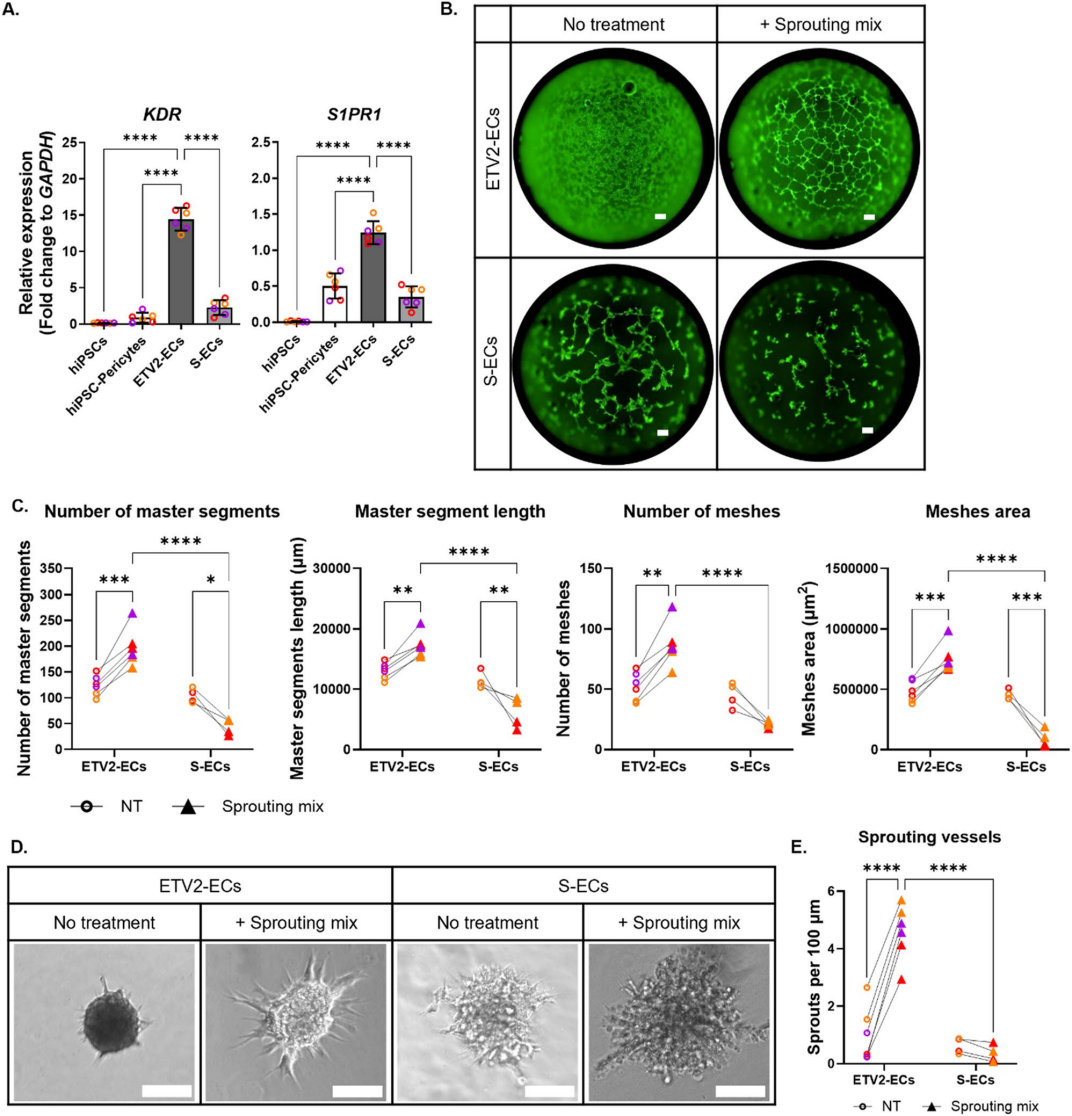
ETV2-ECs Respond to angiogenic stimuli by forming vessel-like structures in both 2D and 3D models. (**A**) Relative mRNA levels of *KDR* and *S1PR1* in hiPSC-pericytes, S-ECs, ETV2-ECs, and hiPSCs., shown as fold change relative to GAPDH. (**B**) Representative 2D tube formation images of S and ETV2-ECs with and without sprouting mix, 6 h post-exposure. Scale bars: 800 μm. (**C**) Quantification of tube formation metrics: number of master segments, master segment lengths, mesh counts, and mesh areas. (**D**) Representative 3D vessel formation images showing sprouting ends formed by S and ETV2-ECs with and without sprouting mix, 24 h post-exposure. Scale bars: 100 μm. (**E**) Quantification of sprouting events, expressed as the number of sprouts per 100 μm of spheroid perimeter in S and ETV2-EC spheroids. Dot plots represent the average of technical replicates for each differentiation batch, color-coded by hiPSC line. Data are presented as mean ± SD. Statistical analysis was performed using two-way ANOVA with Bonferroni’s multiple comparison test. Significance levels are denoted as *p < 0.05, **p < 0.01, ***p < 0.001, ****p < 0.0001

Angiogenesis is a critical function of brain ECs, prompting us to examine the expression of *S1PR1*(Sphingosine-1-Phosphate Receptor 1) and *KDR* (Kinase Insert Domain Receptor, also known as VEGFR2), the receptors for two key angiogenic factors S1P and VEGFA, respectively. Our results showed that *S1PR1* and *KDR* expression levels were significantly higher in ETV2-ECs compared to S-ECs, with lower expression levels also observed in hiPSCs, and hiPSC-pericytes (Fig. 3A). To assess the impact of these changes on EC function, we conducted a 2D tube formation assay with or without the sprouting mix containing VEGFA, S1P, bFGF, and PMA. Our results demonstrated that in the absence of angiogenic stimulation, ETV2-ECs did not form tube-like structures, whereas S-ECs formed simple structures. Surprisingly, after introducing the sprouting mix, ETV2-ECs developed complex tube-like structures, while S-ECs shrank and failed to form any (Fig. 3B). The quantitative analysis assessing the number of segments, segment lengths, number of meshes, and mesh areas confirmed that ETV2-ECs formed more complex structures than S-ECs after being exposed to the sprouting mix (Fig. 3C).

Although the 2D tube formation assay indicated that only ETV2-ECs responded to angiogenic stimuli, the resulting structures were simply linear alignments of cells in 2D, limiting their biological relevance. Therefore, we decided to carry out a 3D vessel formation assay to determine if analogous results were observed. As anticipated, in the absence of a sprouting mix, embedded spheroids of both S and ETV2-ECs failed to demonstrate any vessel-like sprouting. However, upon application of the sprouting mix, ETV2-EC spheroids formed numerous vessel-like sprouts, whereas S-ECs did not (Fig. 3D). We quantified the number of sprouts per 100 μm perimeter of the spheroid to statistically illustrate these differences (Fig. 3E). Although S-ECs formed simple structures in the 2D tube formation assay, their morphology appeared abnormal and deteriorated after exposure to the sprouting mix, suggesting potential cell death. To distinguish between live and dead cells following the 3D vessel formation assay, we conducted propidium iodide (PI) and Hoechst staining. However, we found no significant difference between S and ETV2 spheroids. Both types of spheroids had dead cells, with even brighter PI staining observed in ETV2 spheroids (Figure S3 A), suggesting that the S-EC failure to form sprouts was not caused by increased cell death. These findings demonstrate that ETV2-ECs can respond to angiogenic signals and form vessel-like structures in both 2D and 3D models. Moreover, they exhibit higher expression of key angiogenesis-related receptors, making them a promising model for angiogenesis research.

### APPswe mutation does not affect ETV2-induced EC differentiation or baseline phenotype

Our results indicated that ETV2-ECs closely resemble in vivo ECs in terms of marker expression and their responses to inflammatory and angiogenic stimuli. Therefore, to investigate the effect of AD-associated APPswe mutation on EC functions, we opted for the ETV2 protocol.

**Fig. 4:**
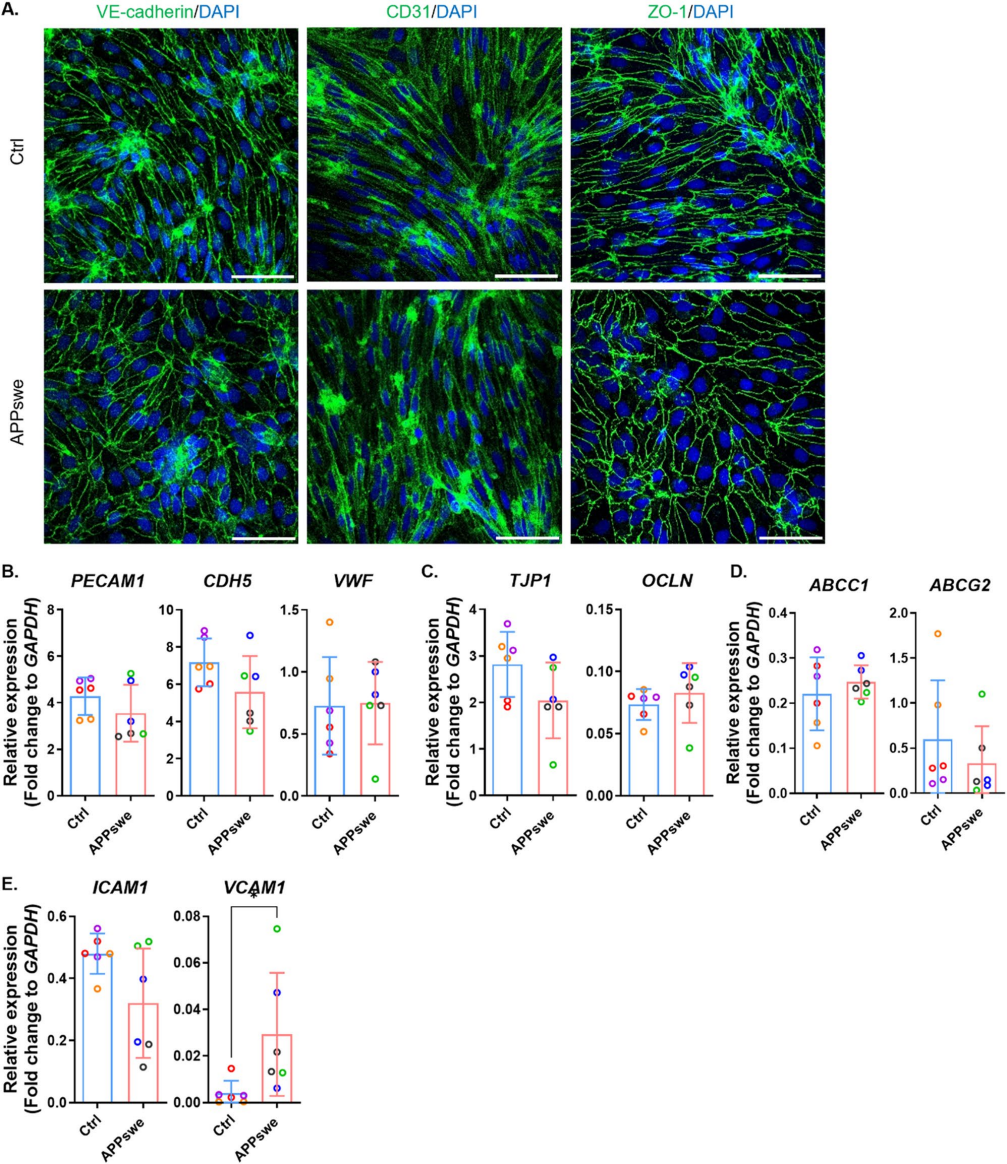
Characterization of ETV2-ECs derived from APPswe Mutant Lines. (**A**) Immunostaining for VE-cadherin, CD31, and ZO-1 in control and APPswe ECs derived from ETV2 protocol. Nuclei are counterstained with DAPI. Scale bars, 50 μm. (**B**-**E**) Relative mRNA expression levels of *PECAM1*, *CDH5*, *VWF* (B), *TJP1*,* OCLN* (**C**), *ABCC1*,* ABCG2* (**D**), *ICAM1*, *VCAM1* (**E**) in control and APPswe ECs. Expression levels are shown as fold change relative to *GAPDH*. Dot plots represent the average of technical replicates for each differentiation batch, color-coded by hiPSC line. Data are presented as mean ± SD. Statistical analysis was performed using two-way ANOVA with Bonferroni’s multiple comparison test. Significance levels are denoted as *p < 0.05, **p < 0.01, ***p < 0.001, ****p < 0.0001

APPswe and Ctrl cells exhibited similar levels of CD31 and VE-cadherin immunoreactivity, as well as similar expression of the TJP ZO-1, indicating that APPswe mutation did not impair hiPSC differentiation to ECs (Fig. 4A). Also, the mRNA levels of EC markers (*PECAM1*, *CDH5*, *VWF*), TJPs (*OCLN*, *TJP1*), and transporters (*ABCC1*, *ABCG2*) were similar in APPswe and Ctrl ECs (Fig. 4B-D). Only *VCAM1*, but not *ICAM1*, showed a significantly higher expression compared to control ECs, while no other differences were observed (Fig. 4E).

### APPswe mutation modulates barrier function and inflammatory responses in ECs

**Fig. 5:**
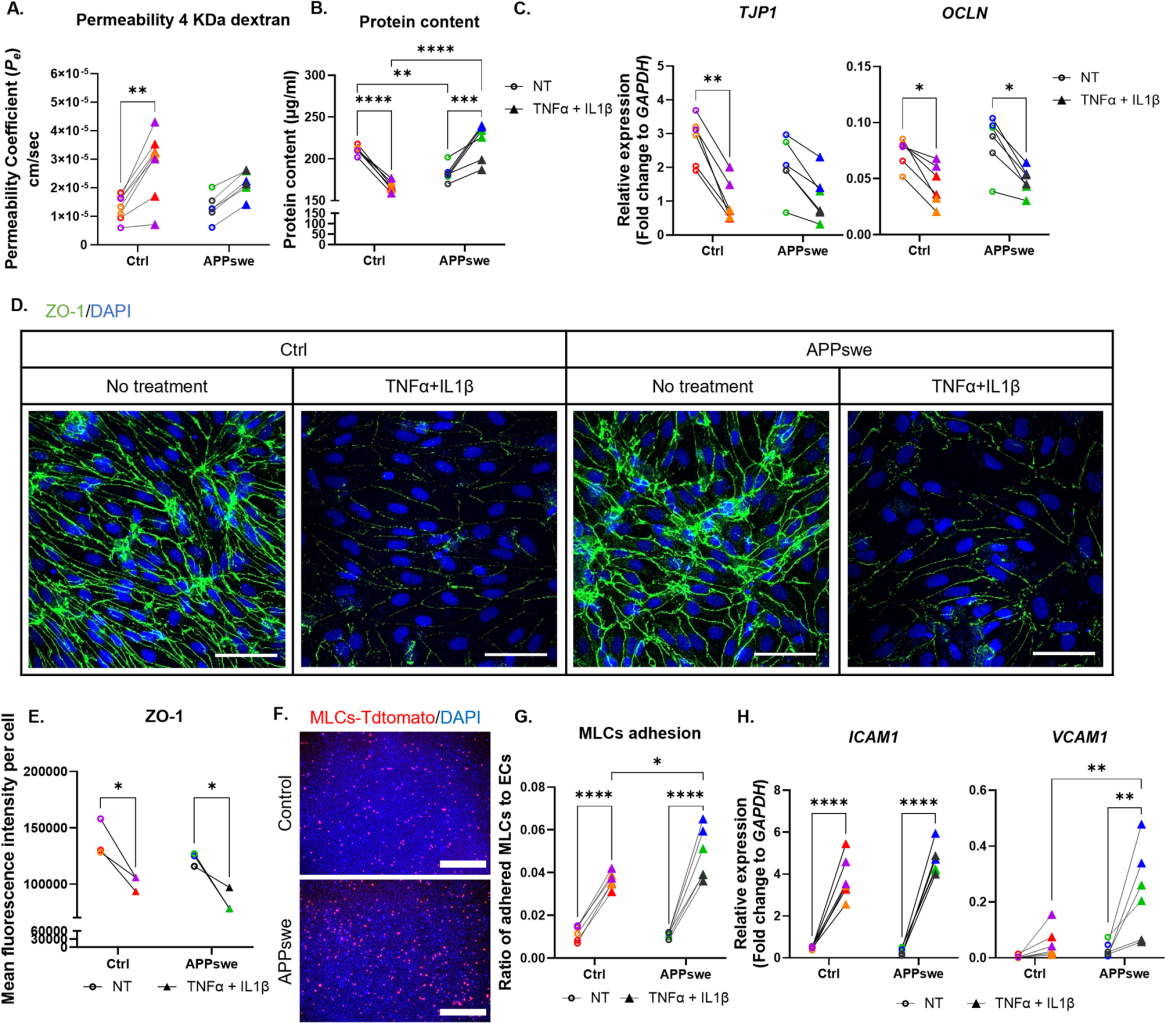
Elevated VCAM1 expression in APPswe ECs drives increased MLC adhesion after inflammation. (A) Endothelial permeability coefficients (Pe) measured using 4 kDa fluorescently labeled dextran in control and APPswe-ECs derived via the ETV2 protocol, before and after exposure to TNFα and IL-1β. (B) Total protein content measured post-assay as an indicator of cell density. (C) Relative mRNA levels of *TJP1* and *OCLN* in control and APPswe ECs before and after cytokine exposure, normalized to *GAPDH* and expressed as fold change. (D) Immunofluorescence staining of ZO-1 in control and APPswe ECs before and after exposure to TNF-α and IL-1β. Nuclei were counterstained with DAPI. Scale bars, 50 μm. (E) Quantification of ZO-1 mean fluorescence intensity per cell, calculated as integrated intensity normalized to cell number. (F) Representative images of DAPI-stained cells after the adhesion assay; red indicates MLCs adhered to control or APPswe-ECs after washing. Scale bars: 750 μm. (G) Quantification of MLC adhesion, calculated as the ratio of adhered MLCs to total DAPI-stained nuclei per field. (H) Relative mRNA levels of *ICAM1* and *VCAM1* in control and APPswe ECs before and after cytokine exposure, normalized to *GAPDH* and expressed as fold change. Dot plots represent the average of technical replicates for each differentiation batch, color-coded by hiPSC line. Data are presented as mean ± SD. Statistical analysis was conducted using two-way ANOVA with Bonferroni’s multiple comparison test. Significance levels are denoted as *p < 0.05, **p < 0.01, ***p < 0.001, ****p < 0.0001.

We then explored permeability changes in APPswe ECs following inflammatory challenge. Surprisingly, control ECs showed on average a greater increase in permeability after cytokine exposure than APPswe ECs (Fig. 5A). Two-way ANOVA revealed a significant effect of cytokine exposure on the expression of *TJP1* and *OCLN*, but no significant genotype effect or interaction was detected in either gene (*TJP1* – treatment: *p* = 0.0001; *OCLN* – treatment: *p* = 0.003; Fig. 5C). Additionally, cytokine exposure increased protein concentration in APPswe EC lysates, possibly due to increased proliferation and/or hypertrophy (Fig. 5B). This may explain the lack of detected permeability changes in APPswe ECs. We further validated the changes in TJP levels by ICC before and after cytokine exposure (Fig. 5D, S4 C). ZO-1 fluorescence intensity was significantly reduced in both control and APPswe ECs following exposure (Fig. 5E (batch1), S4 B (batch2)). However, consistent with the qPCR results, no significant differences were observed between control and APPswe ECs (Figs. 5E, S4 B-C). We also discovered that the APPswe mutation enhanced MLC adherence to cytokine-stimulated ECs (Fig. 5F-G). We examined the expression of LAMs before and after cytokine exposure. Our results showed that TNFα and IL1β exposure strongly induced *ICAM1* expression irrespective of the EC genotype. In contrast, *VCAM1* expression was induced significantly more in APPswe ECs than control ECs (Fig. 5H).

### APPswe ECs exhibited impaired vessel-like structure formation following sprouting mix exposure in both 2D and 3D models

**Fig. 6:**
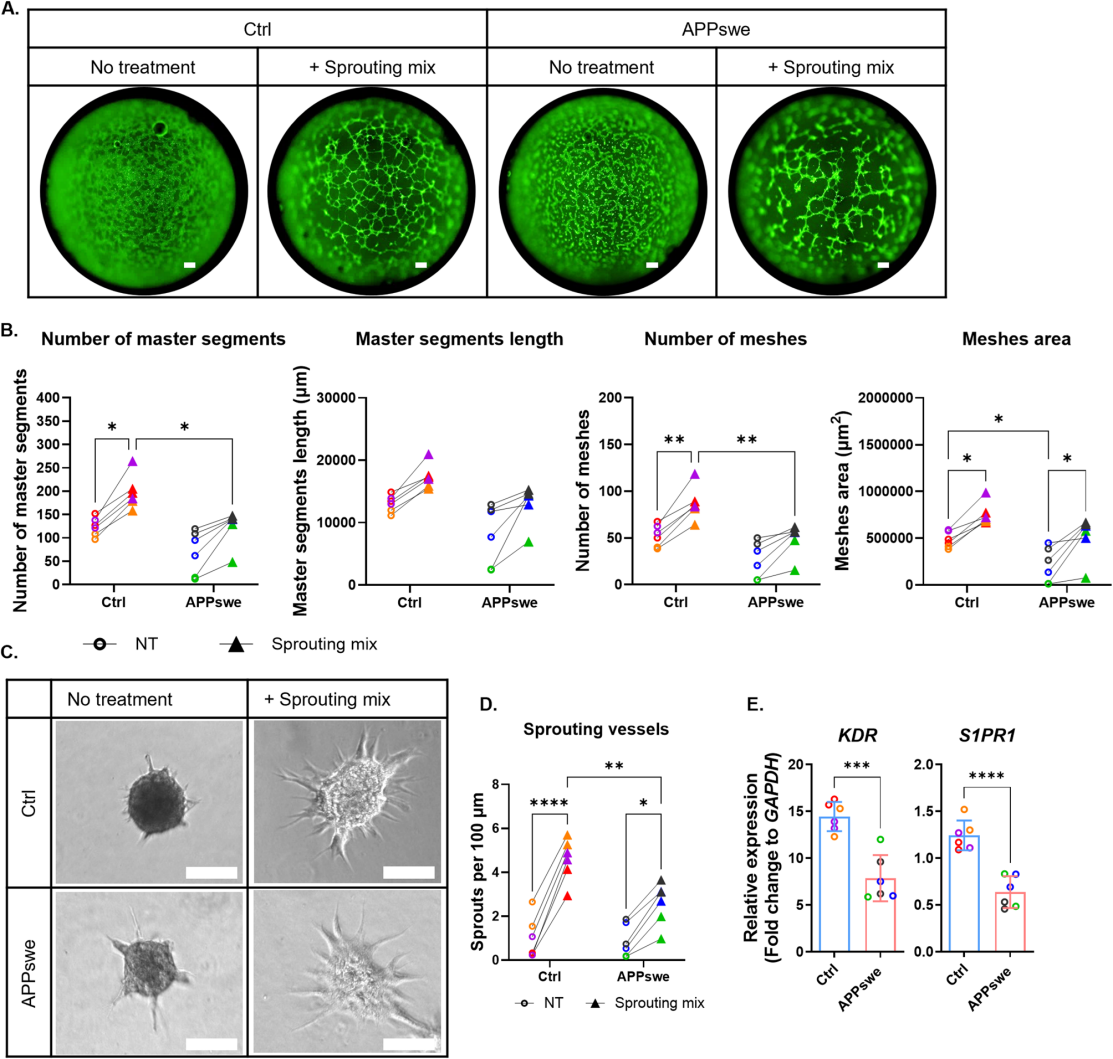
Reduced vessel-like structure formation in APPswe ECs following sprouting mix stimulation. (A) Representative 2D tube formation images of control and APPswe ECs with and without sprouting mix, 6 h post-exposure. Scale bars: 800 μm. (B) Quantification of tube formation metrics: number of master segments, master segment lengths, mesh counts, and mesh areas. (C) Representative 3D vessel formation images showing sprouting ends formed by control and APPswe ECs with and without sprouting mix, 24 h post-exposure. Scale bars: 100 μm. (D) Quantification of sprouting events, expressed as the number of sprouts per 100 μm of spheroid perimeter in control and APPswe EC spheroids. (E) Relative mRNA levels of *KDR* and *S1PR1* in control and APPswe ECs, normalized to *GAPDH* and expressed as fold change. Dot plots represent the average of technical replicates for each differentiation batch, color-coded by hiPSC line. Data are presented as mean ± SD. Statistical analysis was performed using two-way ANOVA with Bonferroni’s multiple comparison test. Significance levels are denoted as *p < 0.05, **p < 0.01, ***p < 0.001, ****p < 0.0001

In the 2D tube formation assay, APPswe ECs tended to exhibit fewer segments and mesh structures compared to control ECs in the absence of the sprouting mix, although this difference was not statistically significant (Fig. 6A-B). The sprouting mix induced less complex tube-like structures in APPswe ECs with significantly fewer segments and meshes than in control ECs (Fig. 6A-B). Further validation using the 3D vessel formation assay revealed a similar effect, with APPswe EC spheroids forming significantly fewer sprouts than control EC spheroids after sprouting mix application (Fig. 6C-D). qPCR analysis confirmed lower expression levels of *KDR* and *S1PR1* in APPswe ECs (Fig. 6E). Thus, the impaired response of APPswe ECs to the sprouting mix containing VEGFA and S1P may be explained by their diminished expression of the relevant receptors.

## Discussion

To verify that temporary overexpression of ETV2 in hiPSCs generates functional ECs, we compared the cells obtained by ETV2 overexpression with a spontaneous differentiation protocol. In accordance with earlier studies [25, 53], we found that ETV2-ECs closely recapitulate the transcriptional and functional characteristics of in vivo ECs. They show high expression of endothelial markers such as *PECAM1* and *CDH5*, TJPs including *TJP1* and *OCLN*, along with angiogenesis-related genes such as *S1PR1* and *KDR*. These genes are associated with key endothelial functions such as cell adhesion, vascular permeability, and angiogenesis. In contrast, the widely adopted spontaneous differentiation (S) protocol yielded a heterogeneous and immature cell population, as indicated by higher proliferation rates (Fig. 2D) and increased expression of epithelial markers such as *KRT8*, and *KRT14* (Figure S2 A). Although previous studies reported detectable levels of ZO-1 (*TJP1*) and CD31 (*PECAM1*) in cells derived by spontaneous protocols [23, 40], we did not observe these markers in our S-ECs generated using similar methods (Fig. 1A).

At the functional level, several assays demonstrated that ETV2-ECs, but not S-ECs, respond robustly to inflammatory and angiogenic stimuli. In the dextran permeability assay, S-EC cultures exhibited lower apparent permeability than ETV2-ECs (Fig. 2B) despite undetectable ZO-1 immunoreactivity. Since total protein content in S-EC cultures was approximately three times higher than in ETV2-ECs (Fig. 2D), we hypothesize that lower permeability of S-ECs may result from increased cell proliferation leading to additional cell layers and greater coverage of Transwell inserts. Notably, both permeability and TJP mRNA and protein expression in ETV2-EC cultures decreased following inflammatory challenge, whereas S-ECs showed no significant response (Figs. 2E and 5D).

Our S-ECs were cultured on Matrigel, which is known to exhibit batch-to-batch variability. Since the spontaneous differentiation protocol relies more heavily on environmental cues than the forced ETV2 overexpression protocol, coating matrix-related factors may partially explain our inability to generate mature S-ECs. Previous studies have shown that maturation on defined matrix coatings (e.g., a mixture of collagen IV and fibronectin) and/or cell sorting can improve the efficiency of EC generation [23]; Nishihara et al., [32, 39, 40]. However, changing the coating matrix requires cell adaptation and cell sorting requires additional equipment and introduces potential variability [51]. Therefore, further optimization of S-EC protocols is needed to improve reproducibility across batches and laboratories. Given that we consistently obtained functional ECs only using the ETV2 overexpression protocol, we selected this approach to study the impact of the APPswe mutation on endothelial cell function.

Vascular dysfunction and increased BBB permeability are commonly observed features in AD patients and mouse models [3, 30, 52]. We did not observe a significant effect of APPswe mutation on the permeability of the EC layer or the expression of TJPs (Fig. 5A–C). However, we did see an increased adherence of MLCs to TNFα/IL1β-stimulated APPswe ECs accompanied by a significantly higher upregulation of *VCAM1* expression in APPswe ECs compared to control ECs. Although we did not directly measure Aβ production in our ECs, earlier studies demonstrated that ECs can produce both APP and different Aβ species [21]. Interestingly, an earlier report demonstrated that soluble Aβ1–40 aggregates increased the adhesion of THP-1 monocyte-like cells to human umbilical vein endothelial cells (HUVECs), suggesting that at least some of the effects we observed in APPswe ECs may be due to increased Aβ production [14].

ICAM1 and VCAM1 are key adhesion molecules that enable the attachment of circulating immune cells to ECs and mediate their infiltration into inflamed tissues [42]. ICAM1 and VCAM1 expression in ECs is typically low under homeostatic conditions but increases during acute and chronic inflammation [27]. There have been conflicting reports regarding the ability of peripheral leukocytes, including neutrophils, monocytes, and T-lymphocytes, to infiltrate brain parenchyma in AD patients and mouse models [4, 37, 57]. However, the accumulation of peripheral leukocytes in the lumen of blood vessels can by itself impair the blood flow, induce EC inflammation, and worsen cognitive functions [11]. The antibodies blocking the interaction of leukocyte integrins with ICAM1 or VCAM1 have shown some promising results in mouse models of AD, including a decrease in microgliosis and improvement in spatial memory function [37, 56, 57]. Interestingly, plasma levels of soluble VCAM1 increase with aging in both humans and mice [9, 56] and positively correlate with cognitive impairment [9, 47], while ICAM1 shows only a mild effect. Thus, an increased adhesion of MLCs to APPswe ECs and an increased expression of *VCAM1* are modeling important pathological aspects of AD that could be further used for drug screening.

AD patients and mouse models display the signs of angiogenesis [5, 44, 50], which is believed to be a compensatory mechanism against cerebral hypoperfusion but may increase the leakiness of the BBB. Also, some recent studies have suggested that angiogenesis in AD is non-productive, resulting in the disassembly of the mature vessels [2, 49]. Our results show that in both 2D and 3D cultures, APPswe ECs formed less complex structures after the application of a sprouting mix, suggesting an impaired response to angiogenic stimuli, likely due to a lower expression of the relevant receptors. Interestingly, earlier cell culture studies have shown that while low (nanomolar) concentrations of exogenous Aβ promote angiogenesis [7], high (micromolar) concentrations vice versa impair angiogenesis [36, 41]. In accordance with our findings, a decrease in *KDR* (*VEGFR2*) expression has been observed in Aβ-treated HUVEC cells, the brain tissue from aged AD transgenic mice [10], and vascular cells isolated from AD patients [49]. In addition, S1PR1 protein has been shown to be downregulated in postmortem human AD brain tissue [8]. These observations support the relevance of our model, indicating that it recapitulates key aspects of in vivo pathology and may serve as a useful system for investigating disease mechanisms and identifying potential therapeutic targets.

In summary, our data provide solid evidence that ETV2-ECs can be used to assess functional changes across different genotypes or following drug exposure. Additionally, they respond effectively to angiogenic and inflammatory stimuli, rendering them an excellent model for future investigations into the involvement of ECs in angiogenesis and inflammation. Furthermore, we provide evidence that AD ECs exhibit transcriptional and functional changes compared to control ECs that may facilitate disease progression and thus could serve as targets for future drug development.

## Conclusions

Combined with results from all functional assays, our findings suggest that ETV2-ECs can serve as a promising model for identifying drug targets in NDDs.

## Supplementary Information

Below is the link to the electronic supplementary material.

Supplementary Material 1

## Data Availability

Any data and materials available for sharing will be provided under a Material Transfer Agreement.

## Abbreviations

AD: Alzheimer’s disease
APPswe: APP Swedish mutation
bFGF: Basic fibroblast growth factor
BBB: Blood-brain barrier
ETV2: E26 transformation-specific (ETS) variant 2
ECs: Endothelial cells
EGF: Epidermal growth factor
E8: Essential 8 Medium
hiPSCs: Human induced pluripotent stem cells
ICC: Immunocytochemistry
IL1β: Interleukin-1 beta
KDR: Kinase Insert Domain Receptor, also known as VEGF receptor 2
LAMs: Leukocyte adhesion molecules
MLCs: Monocyte-like cells
PMA: Phorbol 12-myristate 13-acetate
qPCR: Quantitative qReal Time-PCR
S1P: Sphingosine-1-phosphate
S1PR1: Sphingosine-1-Phosphate Receptor 1
TJPs: Tight junction proteins
TNFα: Tumour necrosis factor alpha
VEGF-A: Vascular endothelial growth factor-A
VWF: Von Willebrand Factor
