# Supplementary material for: APPswe mutation causes functional deficits in endothelial cells generated by transient ETV2 overexpression in human iPSCs
Source: Fluids Barriers CNS. 2025 Nov 21;22:118. doi: 10.1186/s12987-025-00728-8 (PMC12639930; doi:10.1186/s12987-025-00728-8)
Supplement: Supplementary file 1 — Supplementary Material 1 [file 12987_2025_728_MOESM1_ESM.docx]

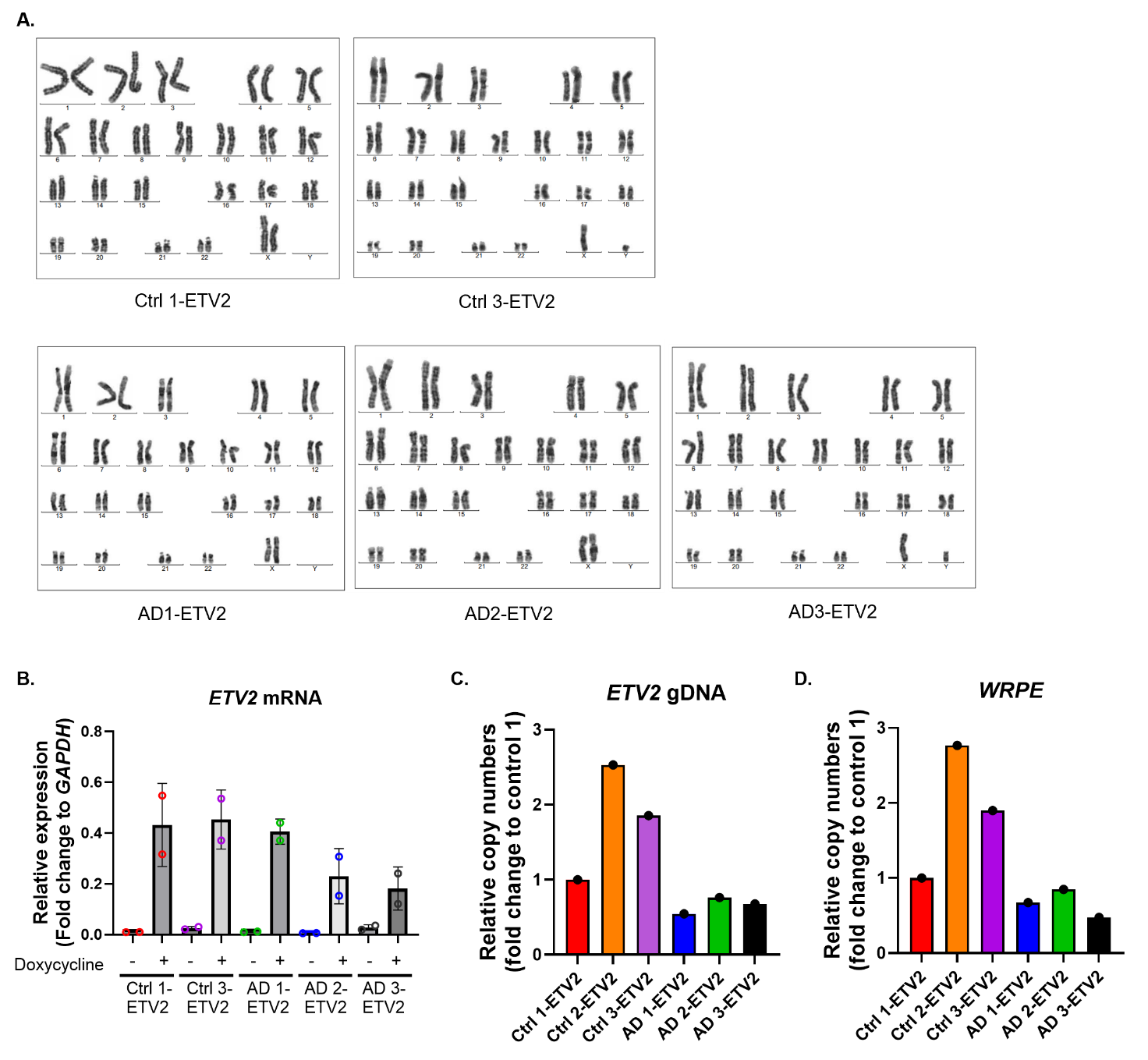


**Supplementary Figure 1. Confirmation of karyotypic integrity and *ETV2* expression in ETV2-transduced lines.**

(A) Representative karyograms from Ctrl1, Ctrl3, AD1, AD2, and AD3 transduced with ETV2 reveal normal euploid karyotypes (46,XX for Ctrl1, AD1, and AD2; 46,XY for Ctrl3 and AD3). (B) *ETV2* expression levels confirmed by qPCR two days after hiPSC lines were exposed to doxycycline or left untreated. Expression levels are normalized to *GAPDH* and expressed as fold change. (C–D) Relative copy numbers of *ETV2* and WRPE sequences in ETV2-transduced lines. Expression levels were normalized to *GAPDH* (used as a single-copy reference in hypoploid cells) and presented as fold change relative to Ctrl 1. Dot plots represent the average of technical replicates for each differentiation batch, color-coded by hiPSC line. Data are presented as mean ± SD.


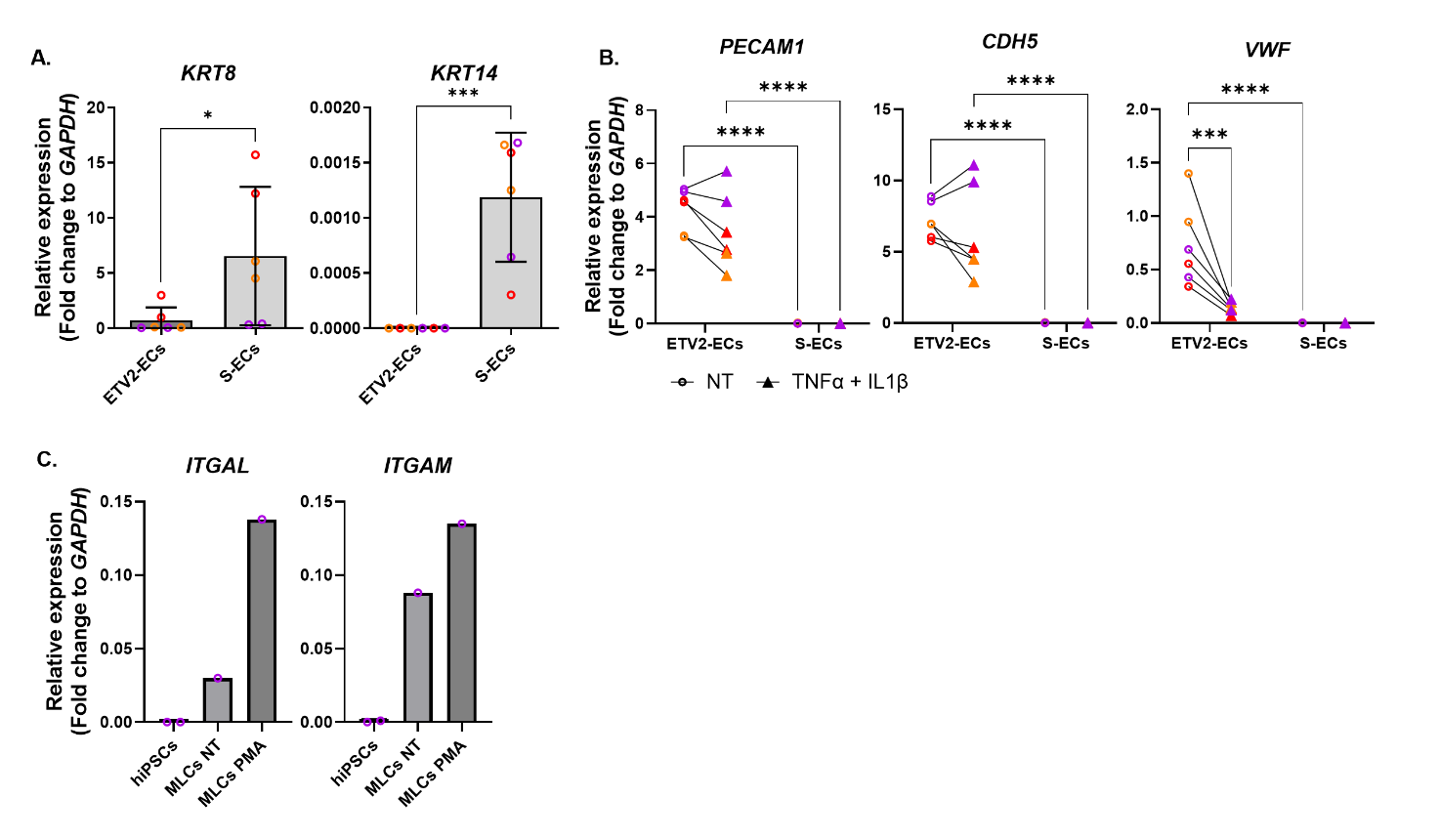


**Supplementary Figure 2.** Changes in EC markers in S and ETV2 ECs before and after inflammatory exposure.

(A) Relative gene expression levels of *KRT8* and *KRT14* in S and ETV2 ECs normalized to *GAPDH* and expressed as fold change. (B) Analysis of relative gene expression levels for *PECAM1*, *CDH5*, and *VWF* in S and ETV2 ECs before and after cytokine exposure, normalized to *GAPDH* and expressed as fold change. (C) Relative gene expression levels of *ITGAL* and *ITGAM* in MLCs before and after 4 hours of PMA exposure, with hiPSCs as controls, presented as fold change relative to GAPDH. Dot plots represent the average of technical replicates for each differentiation batch, color-coded by hiPSC line. Data are presented as mean ± SD. Statistical analysis was performed using two-way ANOVA with Bonferroni’s multiple comparison test. Significance levels are denoted as *p < 0.05, **p < 0.01, ***p < 0.001, ****p < 0.0001.


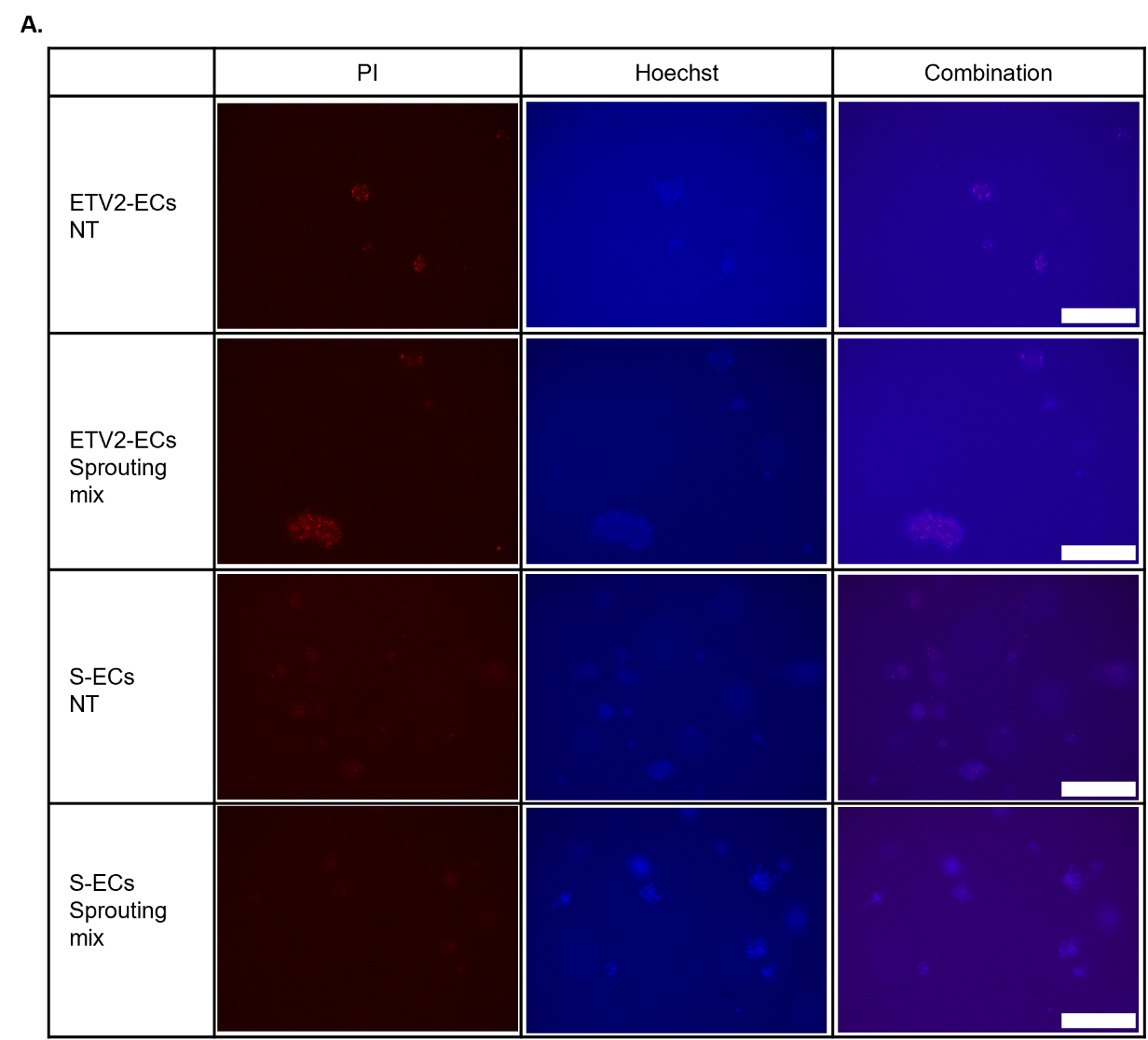


**Supplementary Figure 3. Distinguishing live and dead cells in S and ETV2 spheroids before and after application of the sprouting mix.**

**(A)** Propidium iodide (PI, red) was used to stain dead cells, while Hoechst (blue) stained both live and dead populations, enabling the distinction between live and dead cells in S and ETV2 spheroids, with or without exposure to sprouting mix. Scale bars: 300 µm.

**
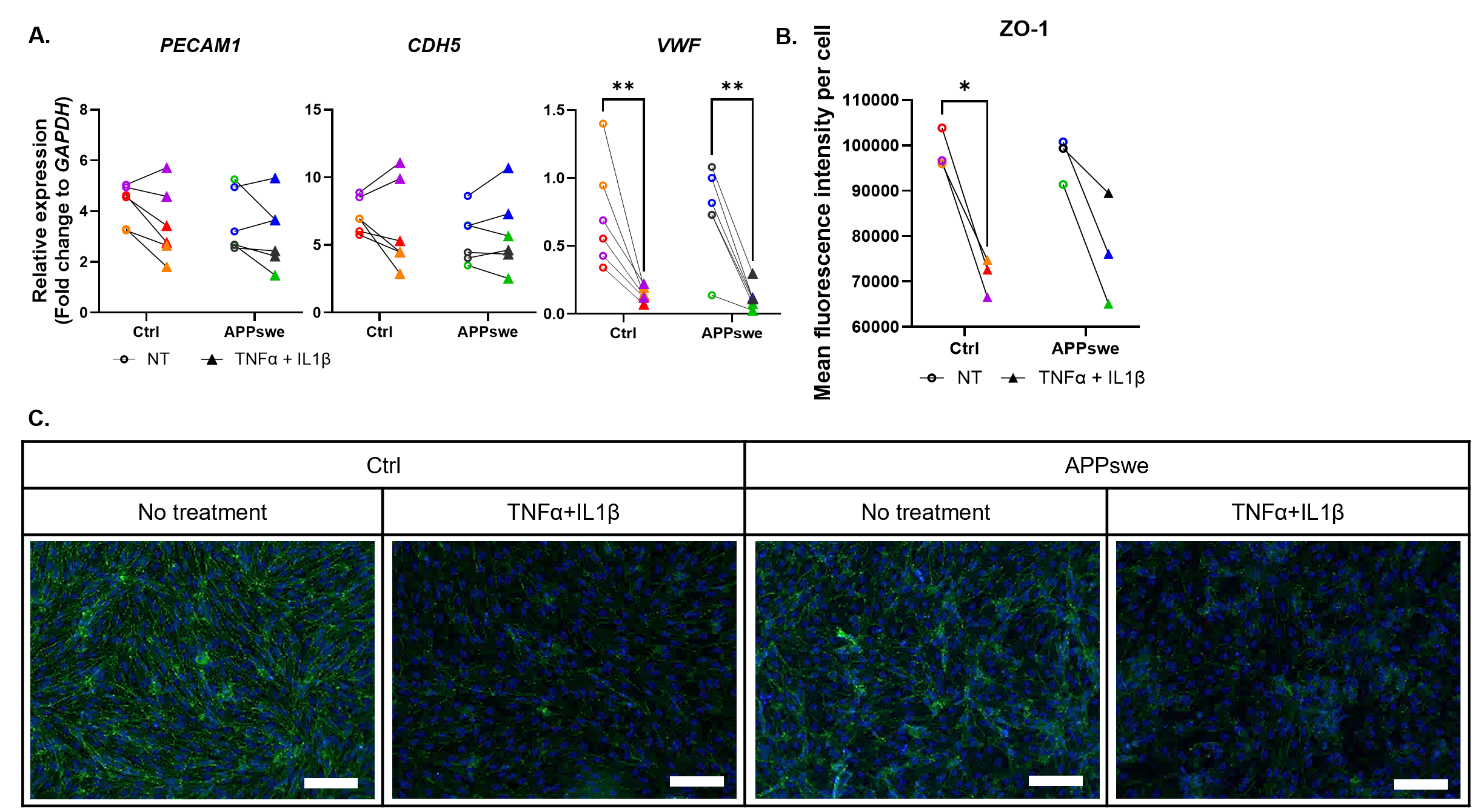
Supplementary Figure S4. Reduction of EC marker and ZO-1 expression in both control and APPswe ETV2-ECs following cytokine exposure**

(A) Analysis of relative gene expression levels for *PECAM1*, *CDH5*, and *VWF* in control and APPswe ECs before and after cytokine exposure, normalized to *GAPDH* and expressed as fold change. (B) Quantification of ZO-1 mean fluorescence intensity per cell, calculated as integrated intensity normalized to cell number. (C) Immunofluorescence staining of ZO-1 in control and APPswe ECs before and after exposure to TNF-α and IL-1β. Nuclei were counterstained with DAPI. Scale bars, 100 μm. Dot plots represent the average of technical replicates for each differentiation batch, color-coded by hiPSC line. Data are presented as mean ± SD. Statistical significance was assessed using student t-test (A-D) or two-way ANOVA with Bonferroni’s multiple comparison test (E), indicated as *p < 0.05, **p < 0.01, ***p < 0.001, ****p < 0.0001.
